# The Association Between Personality Traits and Psychiatric Disorders: A Comparative Study of Violent and Non-Violent Offenders in a Nigerian Correctional Centre

**DOI:** 10.1192/bjo.2025.10188

**Published:** 2025-06-20

**Authors:** Ifedayo Ajayi

**Affiliations:** Royal Cornhill Hospital (NHS Grampian), Aberdeen, United Kingdom

## Abstract

**Aims:** The study examined and compared the association between personality traits and psychiatric disorders among violent and non-violent offenders in a correctional centre in Nigeria.

**Methods:** This cross-sectional comparative study involved 268 participants, evenly divided between violent and non-violent offenders from the Nigerian Correctional Centre in Abeokuta, Ogun State, Nigeria. All consenting participants completed a sociodemographic questionnaire, the Big Five Personality Inventory (BFI-44) to assess personality traits (extraversion, agreeableness, conscientiousness, neuroticism, and openness), and the Mini-International Neuropsychiatric Interview Version 7 (MINI 7.0) to identify psychiatric disorders. The Chi-Square test (χ²) and Fisher’s exact test (FET) were used to determine statistical significance and associations between personality traits and psychiatric disorders with p-value set at <0.05 and confidence interval of 95%. Ethical approval was granted by the Health Research and Ethics Committee of the Neuropsychiatric Hospital Aro, Abeokuta, Ogun State.

**Results:** Openness was the only personality trait with a statistically significant difference between the two offender groups. Violent offenders exhibited a higher proportion of both low (18.7%) and high (17.9%) scores compared with non-violent offenders (11.9% and 9.7%, respectively) (χ²=7.351, p=0.025).

The prevalence of any lifetime psychiatric disorder was significantly higher among violent offenders (35.8%) than non-violent offenders (21.6%) (χ²=0.84, p=0.001). No significant association was found between any personality traits and Depression, Anxiety, Bipolar Affective Disorder, or Antisocial Personality Disorder in both offender groups.

Among violent offenders, low Openness scores were associated with current psychotic disorders (FET=5.57, p=0.039). In contrast, among non-violent offenders, average and high Neuroticism scores were linked to current psychotic disorders (FET=6.51, p=0.022), while low and high Conscientiousness scores were associated with lifetime psychotic disorders (FET=12.15, p=0.002).

Furthermore, among non-violent offenders, low Agreeableness scores were associated with alcohol use disorder in the past 12 months (FET=7.28, p=0.014), while high Openness scores were linked to substance use disorders in the past 12 months (FET=6.29, p=0.041).

**Conclusion:** The study revealed that the association between personality traits and psychiatric disorders varies between violent and non-violent offenders. Although this adds modestly to existing knowledge, larger studies are necessary to generate more robust findings.

